# Trapping a Hot Drop on a Superhydrophobic Surface with Rapid Condensation or Microtexture Melting

**DOI:** 10.3390/mi9110566

**Published:** 2018-11-02

**Authors:** Samira Shiri, Armela Murrizi, James C. Bird

**Affiliations:** Department of Mechanical Engineering, Boston University, Boston, MA 02215, USA; amurrizi@bu.edu

**Keywords:** Nasturtium leaf, smart superhydrophobic surface, hot drop, condensation, microtexture melting

## Abstract

A water drop can bounce upon impacting a superhydrophobic surface. However, on certain superhydrophobic surfaces, a water drop will stick rather than bounce if it is sufficiently hot. Here, we aim to better understand the mechanisms that can lead to this bouncing-sticking transition. Specifically, we model two potential mechanisms in which a superhydrophobic surface could trap a sufficiently hot drop within milliseconds: melting of microtextured wax and condensation of the vapor within the superhydrophobic texture. We then test these mechanisms through systematic drop impact experiments in which we independently vary the substrate and drop temperatures on a waxy superhydrophobic Nasturtium leaf. We find that, whenever the surface or the drop is above a microtexture-melting temperature, the drop sticks. Below this temperature, a critical temperature threshold for bouncing can be predicted and controlled by considering the relative timescales between condensation growth and drop residence time. We envision that these results can provide insight into the design of a new class of superhydrophobic surfaces to act as a rapid thermal fuse to prevent drops that exceed a critical temperature from bouncing onto a thermally sensitive target.

## 1. Introduction

**Figure 1:**
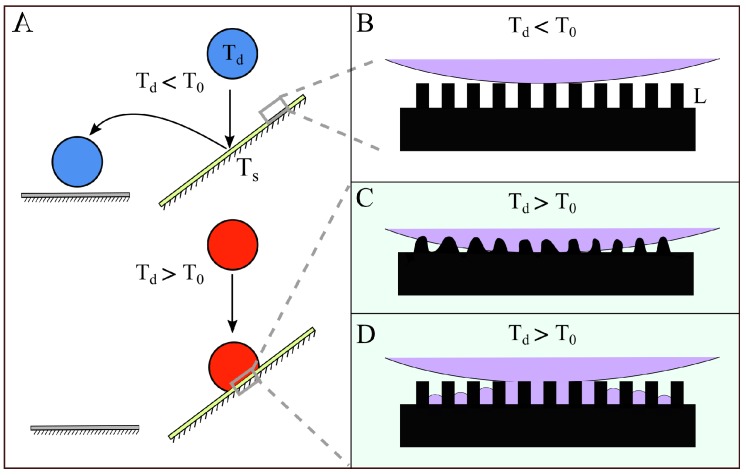
A schematic illustrating how a thermally responsive superhydrophobic material could act as a fuse to prevent water above a critical temperature from reaching a sensitive surface. (**A**) If the superhydrophobic surface that is at $T_{s}$ rapidly pinned drops above a critical temperature $T_{0}$, then a water drop with temperature $T_{d}$ below $T_{0}$ (top) would bounce off of the surface onto the target, whereas a water drop with temperature $T_{d}$ above $T_{0}$ (bottom) would stick, protecting the target; (**B**) surface microtexture with lengthscale *L* can trap air beneath the drop (Cassie–Baxter state), enabling the drop to bounce; (**C**) If heat from the drop melts the microstructure, the drop could enter a Wenzel state and stick; (**D**) Alternatively, if liquid from the drop evaporates and condenses within the microstructure, the drop could also enter a Wenzel state and stick.

For most surfaces, an impacting water drop will stick upon contact; however, for a superhydrophobic surface, an impacting water drop can spread out and recoil to such an extreme that it can completely bounce off of the surface [1,2,3]. The degree of superhydrophobicity can be tuned by modulating the chemistry and structure of the surface, thus enabling external control of whether a particular drop bounces or sticks [4,5,6]. For example, by modulating the superhydrophobic properties of the surface, a microdevice could be designed to prevent drops above a critical temperature from reaching a thermally sensitive region (Figure 1). A challenge with using external stimulants to modulate such a device is that the control would likely involve separate sensing, processing, and actuating steps. However, if a superhydrophobic surface can be designed to passively adjust its functionality in response to a drop property, then it can be used as a sensor or fuse. Here, we explore how one might design a smart superhydrophobic surface in which the surface can sense the drop temperature and act within milliseconds to selectively trap a drop that exceeds a critical threshold. In particular, we identify thermally-induced sticking mechanisms and predict the conditions that would be needed for them to act faster than the time it takes for the drop to bounce off the surface. A superhydrophobic surface combines chemical hydrophobicity with microscopic texture so that, in the Cassie–Baxter state, a water drop resides on top of the air-filled microtexture (Figure 1B) [7]. The air under the drop leads to a large effective contact angle and low contact friction, a combination that can enable the drop to bounce. By contrast, if the drop enters a Wenzel state, the water drop permeates the microtexture [8]. This attachment of water with surface roughness dampens the recoil following impact so that the drop sticks to the surface. Whether a drop adopts the Cassie–Baxter state or the Wenzel state strongly depends on the microtexture geometry and surface chemistry, as well as the surface tension of the liquid [9]. Indeed, attempts to create surfaces that can repel scalding water have been frustrated in part by drop sticking, a result that has been largely attributed to the lower surface tension associated with the hotter water [10].

There is evidence that surface tension is not the only mechanism by which a sufficiently hot drop could initiate a Cassie–Baxter to Wenzel state transition. Liu et al. [10] found that drops would stick to lotus leaves when the drop temperature exceeded $55{\;}^{\circ }$C and speculated that the waxy microtexture could be melting at these temperatures [10] (Figure 1C). Alternatively, if the surface is below the dew point, water vapor from the air can condense within the microtexture and drive the drop into a Wenzel state [11,12]. Even if the surface is above the dewpoint of the ambient air, the slight evaporation of the water drop residing on the microtexture can locally saturate the air, which can then condense on a slightly cooler microtexture (Figure 1D). In fact, experiments of static drops resting on superhydrophobic surfaces have shown that, when the surface temperature is lower than the drop temperature, then this evaporation–condensation process can transition the drop into a Wenzel state [13].

Past studies that have identified microtexture melting or evaporatation-condensation as processes that could initiate a Cassie–Baxter to Wenzel state transition have generally considered applications in which this transition is undesirable. In contrast, we focus on exploiting the transition, appreciating that these mechanisms must sufficiently modify the surface in a short enough time to trap the drop before it bounces. The time that a drop is in contact with a superhydrophobic surface is predominantly dictated by the inertia and surface tension of the drop with some additional tuning possible by exploiting the texture or geometry of the surface [14,15,16,17,18,19]. For a millimeter-sized water drop, this contact time is on the order of 10 ms, which can be short enough to limit certain transport properties such as conductive heat exchange. Indeed, for these sized drops, it is expected that, if a drop were to bounce rather than stick, only around 1% of the heat would be transferred [20]. However, it is unclear under what conditions this heat or any evaporation and condensation would modify the surface significantly enough to trap the drop. To address this question, we combine experiments and modeling. We carry out drop impact experiments on Nasturtium leaves at different temperatures and compare the results to models in which we estimate the time it would take to change the surface conditions relative to the time the drop takes to bounce.

## 2. Materials and Methods

**Figure 2:**
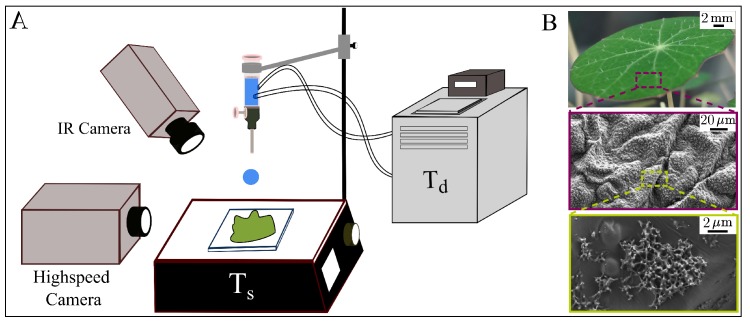
(**A**) a schematic of the experimental set. Cameras film a water drop at temperature $T_{d}$ as it falls from a suspended needle and impacts a Nasturtium leaf heated to temperature $T_{s}$; (**B**) images of a Nasturtium leaf taken at different magnifications illustrate the hierarchical microstructure.

To explore the physics underlying the bouncing-sticking transition of a drop to a superhydrophobic surface, we carry out a series of experiments in which both drop and surface temperature are varied systematically. For each experiment, a single drop of water is released from a suspended needle, falls, and impacts a superhydrophobic Nasturtium leaf. The drop temperature $T_{d}$ is controlled with a water bath, and temperature of the leaf surface $T_{s}$ is controlled using a hot plate. To ensure the impact dynamics between experiments are similar, the height and gauge of the suspended needle are fixed to maintain a radius of R≈1mm and impact velocity of $V\approx 0.75\;{\mathrm{ms}}^{-1}$. The subsequent impact dynamics are recorded simultaneously with high speed and thermal cameras. High speed images are captured with a Photron Fastcame SA-X2 (made in Japan) at a frame rate of 8000 frames per second and a 200mm Nikon lens (made in Japan). Thermographic images are simultaneously recorded from an inclined perspective using an FLIR A655sc thermal camera (made in Sweden) at frame rate of 200 frames per second with a close-up infrared (IR) camera lens (Figure 2A). These thermographic images are used to calculate $T_{d}$ and $T_{s}$.

Nasturtium leaves are ideal for these experiments for three reasons. First, these leaves are naturally superhydrophobic due to a waxy microstructure that can melt at temperatures below the boiling-point of water. Second, we find that condensation can form within the microstructure when the surface is cooled below the dew point. Finally, these plants are relatively simple to grow within in the lab, providing a reliable source of waxy superhydrophobic material. Prior to an experiment, a leaf is cut from the Nasturtium plant, secured to a glass slide, and placed on the hot plate. Images of one of our leaves reveal the shape and hierarchical surface structure responsible for the Nasturtium superhydrophobicity (Figure 2B).

## 3. Results

**Figure 3:**
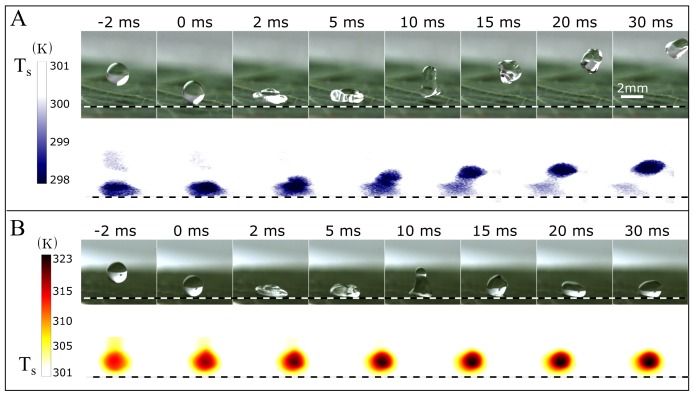
A superhydrophobic Nasturtium leaf that lets a cold drop bounce off of its surface traps a hot drop. (**A**) A cold water drop at temperatures 298K impacts a Nasturtium leaf that is initially at ambient temperature 301K. High-speed images show that the cold water drop spreads, retracts and leaves the surface at a finite time 10ms. Simultaneously, thermal images show that the drop is at the lower temperature than the leaf in this experiment; (**B**) a hot drop at temperature 323K at the same impact condition sticks to a Nasturtium leaf that is initially at ambient temperature 301K. Simultaneous thermographic images show a temperature map of the drop and substrate during impact. The dotted line shows the contact line between drop and leaf.

The interaction dynamics between a drop and Nasturtium leaf during an impact is demonstrated experimentally in Figure 3. In the first set of images (Figure 3A), a chilled water drop with radius R=1 mm and temperature $T_{d}=298\;K$ (25${\;}^{\circ }$C) falls and impacts a surface that is at room-temperature and measured to be $T_{d}=301\;K$. The impact dynamics are captured simultaneously with both a high-speed from the side and thermal camera from an inclined perspective. The high speed images shows that, when the chilled drop contacts the superhydrophobic surface, it bounces. The time that the drop is in contact with surface, defined as the contact or residence time $t_{r}$, is approximately 10ms. Thermal images of this bounce highlight that the drop is at a lower temperature than the leaf. In the second set of images (Figure 3B), a hot water drop with temperature $T_{d}=323\;K$ falls onto a Nasturtium leaf under nearly identical impact conditions. High speed images reveal that the hot drop is trapped by the leaf and sticks to it. Thermal images of the impact confirm that the drop is hotter than the ambient-temperature leaf.

**Figure 4:**
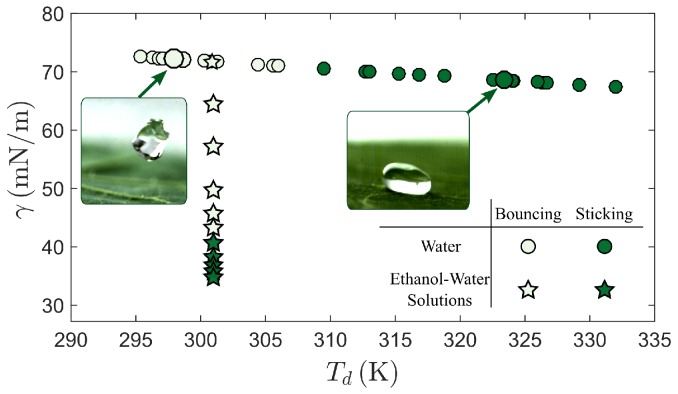
Effect of surface tension γ and drop temperature $T_{d}$ on the bouncing-sticking transition for a Nasterium leaf at ambient conditions. For water drops, the transition between bouncing (open circles) and sticking (filled circles) occurs when the drop temperature $T_{d}\approx 307\;K$, which corresponds to a surface tension γ≈70mN/m. For drops of varying ethanol-water concentrations at ambient conditions, the transition between bouncing (open stars) and sticking (filled stars) occurs when the surface tension is at γ≈43mN/m. This difference in surface tension suggests that surface tension alone cannot account for the transition. Note that the larger circles correspond to the specific drop illustrated in Figure 3.

To evaluate the role of temperature in the trapping process, we extend the experiments highlighted in Figure 3 to a variety of drop temperatures that range from $T_{d}=295\;K$ to 332K. Water drops that are colder than a threshold temperature of approximately 307K bounce off of the surface (Figure 4, open circles), whereas water drops hotter than that threshold temperature stick to the surface (Figure 4, filled circles). The surface tension of many liquids, including water, decreases with increasing temperature [21]. This relationship can be approximated as $\gamma \left(T_{d}\right)=75.7-0.14T_{d}$, where $\gamma (T_{d})$ is the surface tension in units of mN/m for a water drop with temperature $T_{d}$ specified in ${}^{\circ }C$ [22]. Thus the surface tension of water drops at the threshold temperature is calculated to be γ≈70mN/m, although it is likely that this surface tension is slightly lower due to natural surfactants that can accumulate on the water interface.

To assess whether this critical temperature can be explained solely as a consequence of a critical surface tension, we compare the threshold surface tension for varying drop temperatures with the threshold surface tension for varying drop compositions (Figure 4). Specifically, we repeat the drop impact experiments at ambient conditions using drops that contain varying concentrations of ethanol and water. Due to the low surface tension of pure ethanol (γ=22mN/m), adding a small amount of ethanol to water can dramatically lower the surface tension. Given that these experiments were carried out at room temperature, we approximate the drop temperatures as remaining constant at T=301K (Figure 4), and estimate the surface tension based on the concentration of ethanol in the drop following the empirical analysis by Khattab et al. [23]. At low ethanol concentrations, the drop bounces off of the Nasturtium leaf (Figure 4, open stars), whereas, at sufficiently high ethanol concentrations, the drop will stick on the surface (Figure 4, filled stars). The surface tension that corresponds to this transition is γ≈43mN/m, a value that is far less than the surface tension of the water, even at the hottest temperatures. Therefore, it appears that the critical temperature for a water drop cannot be explained solely as a consequence of a critical surface tension, and instead relies on a mechanism in which the temperature modifies the surface during contact.

**Figure 5:**
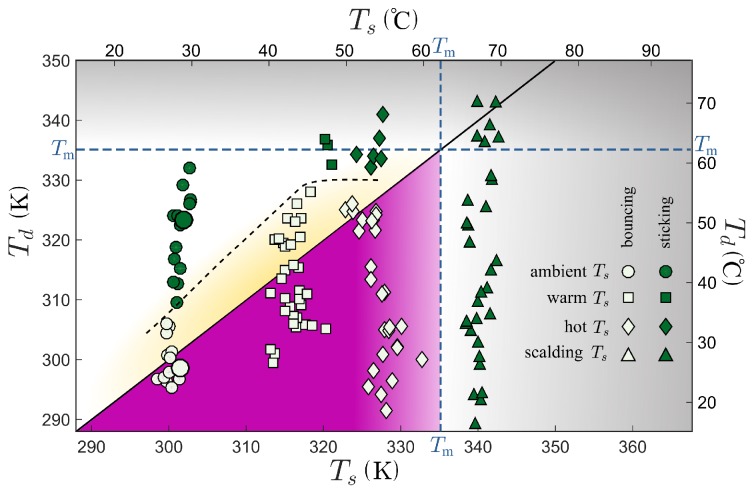
Effect of drop temperature $T_{d}$ and surface temperature $T_{s}$ on the bouncing-sticking transition on the leaf. Surface temperatures are separated into four groups for comparison: ambient (circles), warm (squares), hot (diamonds), and scalding (triangles). The transition between drop bouncing (open symbol) and sticking (closed symbol) is identified by a dashed line as a guide for the eye. The shaded gray region indicates temperatures above a surface melting temperature $T_{m}$. Below these temperatures, the purple region denotes where drops are colder than the surface, and the lighter yellow region highlights where drops bounce despite being warmer than the surface.

Given the importance of surface temperature in both the microtexture melting mechanism and the evaporation–condensation sticking mechanism, experiments with the water at varying drop temperatures are repeated at higher surface temperatures by placing the leaf on a hotplate. The drop release height and drop size continue to be fixed to maintain nearly identical impact conditions. Figure 5 illustrates the results for ambient (circles), warm (squares), hot (diamonds), and scalding (triangles) surface temperatures. Here, we categorize the surface temperature as warm when it is between $T_{s}=313\;K$ and 321K, hot when it is between 322K and 333K, and scalding when it is above 336K. When the surface is heated above $T_{s}\approx 335\;K$, the leaf begins to visibly deform and the drops stick for all drop temperatures $T_{d}$ (gray region). These results suggest that the surface geometry or chemistry could be changing, and we specify this transition as a microtexture melting temperature $T_{m}$. At lower temperatures, the results show that, when a water drop is cooler than the surface (purple area), the drop always bounces off of the surface. However, when the drop is hotter than surface, it may either bounce off (yellow region) or stick to the surface (white region). The drop temperature $T_{d}$ associated with the bouncing-sticking transition (denoted by a dashed guideline in Figure 5) increases with the surface temperature $T_{s}$ before appearing to level-off as the drop temperature approaches $T_{m}$.

## 4. Discussion

The results demonstrate that a Nasturtium leaf, and presumably any similar superhydrophobic surface, can selectively trap water drops that exceed a critical temperature while permitting colder drops to bounce. Furthermore, the experiments indicate that this phenomenon cannot be accounted for by a reduction surface tension alone. Specifically, the results show that a hot drop would stick on the superhydrophobic surface when, at the same surface tension, an ambient-temperature drop would bounce (Figure 4). Additionally, a heated drop that would stick on an ambient-temperature surface might bounce had the surface been heated (for example $T_{d}=320$ K in Figure 5). Thus, the temperature of the drop and the surface both appear to be important parameters for the bouncing-sticking transition, independent of the surface tension. This result is noteworthy because it suggests that the temperature of the drop can significantly affect the properties of the superhydrophobic surface. Because the drop and surface only interact during the bounce, the mechanism required to stop a bounce must sufficiently modify the surface in the short time before the bounce is complete.

Drawing from past studies, we focus on two alternative mechanisms: melting of surface microtexture and evaporation–condensation within the superhydrophobic texture. The results in Figure 5 might appear inconsistent with both mechanisms. Specifically, if a drop at $T_{d}=310\;K$ were able to melt the microtexture, then it would seem improbable that drops of any temperature would bounce if the surface were above this temperature $T_{s}=310\;K$, yet the results illustrate that they do (thus we estimate $T_{m}\approx 335\;K$). Similarly, the existence of the yellow region in Figure 5 is in contrast with previous works for static drops, which predicts sticking for a drop whenever its temperature is larger than that of the surface [13]. However, if the dynamics are considered, it might be possible that, depending on the conditions, there could be regimes in which these effects are present but insufficient to inhibit bouncing.

### 4.1. Melting of the Surface Microtexture

The height of surface microtexture is one of the physical conditions that can noticeably influence transition from the Cassie–Baxter state (necessary for drop bouncing) to the Wenzel state [9]. When the drop temperature is larger than the melting point of microtexture material, melting may shorten and smooth the microtexture surface and thus create conditions that promote the Wenzel state (Figure 1C). To model this melting process, we approximate the superhydrophobic surface as a semi-infinite body that undergoes phase change. We assume a uniform solid surface temperature $T_{s}$ that is cooler the material melting point $T_{m}$. Subsequently, at t=0, the drop contacts the superhydrophobic surface and resides on the surface over the period of the residence time $t_{r}$. If the drop temperature is cooler the substrate melting point ($T_{d}<T_{m}$), then the substrate remains intact and the drop bounces. However, if the drop temperature exceeds the substrate melting point ($T_{d}>T_{m}$), the energy transfer can induce a phase change. The amount of material that can melt will grow with the amount of time that that heat can transfer from the hot drop. The extent of this melting can be estimated by the position of the self-similar melting front $x_{m}$, which we refer to as the melted length. Thus, this moving boundary problem can be solved by using Neumann’s solution for the melting of a semi-infinite body [24].

Based on Neumann’s problem, when a solid–liquid interface forms as a result of melting, two regions can be defined that obey the following governing equations: a liquid region ($0<x<x_{m}$) where $\frac{\partial^{2}T_{\ell }}{\partial x^{2}}=\frac{1}{\alpha_{\ell }}\frac{\partial T_{\ell }}{\partial t}$ and a solid region ($x_{m}<x$) where $\frac{\partial^{2}T_{S}}{\partial x^{2}}=\frac{1}{\alpha_{S}}\frac{\partial T_{S}}{\partial t}$. Applying the boundary conditions $T_{\ell }\left(0,t\right)=T_{d}$ and $T_{\ell }\left(x_{m},t\right)=T_{m}$ for the liquid, $T_{S}\left(x_{m},t\right)=T_{m}$ and $T_{S}\left(\infty ,t\right)=T_{s}$ and the solid, and the initial conditions $T_{S}\left(x,0\right)=T_{s}$, $x_{m}\left(t=0\right)=0$ together with the interface energy equation, leads to:

(1) $$\frac{exp(-\lambda^{2})}{erf(\lambda )}-\sqrt{\frac{\alpha_{\ell }}{\alpha_{S}}}\frac{\kappa_{S}}{\kappa_{\ell }}\frac{T_{m}-T_{s}}{T_{d}-T_{m}}\frac{exp(-\alpha_{\ell }\lambda^{2}/\alpha_{S})}{1-erf(\sqrt{\alpha_{\ell }/\alpha_{S}})\lambda }=\frac{\sqrt{\pi }L\lambda }{c_{\ell }\left(T_{d}-T_{m}\right)}.$$

Here, the subscripts *ℓ* and S denote the properties of the liquid and solid phase, respectively, κ is the thermal conductivity, *c* is the thermal capacity, α is the thermal diffusivity, ρ is the density and L is the latent heat of fusion. This equation provides a value of λ that can subsequently be used to calculate the melted length, noting $x_{m}∼\lambda \sqrt{\alpha_{\ell }t_{r}}$. Therefore, if melting occurs over the entire time that the drop resides on the surface ($t_{r}$), the length of surface microtexture would be $L-x_{m}$, which—depending on conditions—could transition the drop from the Cassie–Baxter to the Wenzel state.

According to the solution of Equation (1), the melting mechanism predicts that a drop would more readily melt the surface if the surface were warmer. Specifically, if melting of the surface microtexture were responsible for the observed bouncing-sticking transition, then the threshold drop temperature would be expected to decrease with increasing surface temperature $T_{s}$. This prediction is in stark contrast to what is observed; the threshold temperature increases with surface temperature, at least up to a point. Thus, it seems unlikely that melting of the microstucure would be responsible for the transition for $T_{d}<T_{m}\approx 335$ K in these experiments (Figure 5).

### 4.2. Condensation of the Vapor within the Superhydrophobic Texture

Condensation is another mechanism that has been attributed to the transition from the Cassie–Baxter to the Wenzel state (Figure 1D). Through this mechanism, liquid evaporates from the drop to saturate the air within the microtexture and then condenses. If air pockets within the microtexture fill with water, then the drop transitions to the Wenzel state. Here, we estimate the timescale associated with this evaporation–condensation process. Specifically, we model the air within the microtexture between the surface and the drop, neglecting any fluxes to regions that are not covered by the drop. Noting that condensation occurs only when the relative humidity is maintained at 100%, we first calculate the timescale for evaporation to fully saturate the air, and then we calculate the timescale for sufficient liquid to condense to fill up the air gap within the microstructure.

To estimate the time needed to saturate the microtexture air, we note that the mass flux across the liquid interface can be modeled as: $J=-D\frac{\partial C}{\partial x}\approx D\frac{C_{s}-C_{\infty }}{L}\approx \frac{DC_{s}}{L}\left(1-R_{H}\right)$. Here, *J* is the evaporation rate, $D=2\times 10^{-5}$ m${}^{2}/$s is the diffusion coefficient of water [25], $C_{s}$ is the concentration of the saturated vapor, $C_{\infty }=C_{s}R_{H}$ is the concentration far from the evaporating liquid, $R_{H}$ is relative humidity and *L* is a characteristic lengthscale on order of the surface microtexture. By integrating the equation with respect to time, the relative humidity can be computed as a function of time: $R_{H}\left(t\right)=1-\left(1-R_{H0}\right)e^{-t/\tau_{e}}$, where $R_{H0}$ is the humidity of the ambient air and $\tau_{e}$ is the characteristic evaporation timescale. The value of $\tau_{e}∼\frac{LV}{DA}=\frac{L^{2}}{D}$ where *V* is the total volume of gap between microstructure and *A* is its projected area. Thus, the time for the air within the microtexture to become saturated relative to the drop residence time is $t_{e}/t_{r}\approx \frac{L^{2}\gamma }{D{\left(\rho R^{3}\right)}^{1/2}}$. In the experiments, the evaporation time is estimated to be on the order of a microsecond. Because this timescale is more than a thousand times faster than the residence time, the air within the microstructure can be estimated as being fully saturated.

We next estimate the condensation time $t_{c}$, which we define as the characteristic time needed for condensate to grow and fill the superhydrophobic microtexture. If saturated air contacts a surface that is at slightly lower temperature, the saturated air will locally cool and condensation will occur. Condensation will continue as long as warmer saturated air is cooled on the surface. To estimate the condensation rate, we adopt a condensation model proposed by Kim et al. [26] in which the rate of condensation growth $\frac{dr}{dt}$ can be approximated as $\frac{dr}{dt}\approx h_{i}\left(\frac{T_{sat}-T_{s}}{\rho_{\ell }\;H_{fg}}\right)$. Here, $h_{i}$ is the interfacial heat transfer coefficient, $T_{sat}$ is the saturated air temperature, $T_{s}$ is the surface temperature, $H_{fg}$ is the latent heat of vaporization and $\rho_{\ell }$ is the water density. This expression can be further reduced by modeling the interfacial heat transfer coefficient $h_{i}=\left(\frac{2\hat{\sigma }}{2-\hat{\sigma }}\right)\left(\frac{\rho_{v}H_{fg}^{2}}{T_{sat}}\right){\left(\frac{\bar{M}}{2\pi \bar{R}T_{sat}}\right)}^{1/2}$ in terms of the vapor properties [27]. Additionally, we estimate the time to fill a microtexture with characteristic lengthscale *L* as $t_{c}\approx L{\left(\frac{dr}{dt}\right)}^{-1}$, so that this time is modeled in terms of the temperatures as:

(2) $$t_{c}\approx \frac{2-\hat{\sigma }}{2\hat{\sigma }}{\left(\frac{2\pi \bar{R}}{\bar{M}}\right)}^{1/2}\left(\frac{\rho_{\ell }L}{\rho_{v}\;H_{fg}}\right)\left(\frac{T_{sat}^{3/2}}{T_{sat}-T_{s}}\right),\;\;\;\;\;T_{sat}=\frac{\left(T_{d}+T_{s}\right)}{2}.$$

Here, $\hat{\sigma }$ is the condensation accommodation coefficient [28], $\bar{R}$ is the universal gas constant, $\bar{M}$ is the the molecular weight, $\rho_{\ell }$ is the liquid density, $\rho_{v}$ is the vapor density, and $T_{sat}$ is temperature of the saturated vapor within the microstructure, which is modeled to be halfway between the drop temperature $T_{d}$ and surface temperature $T_{s}$.

**Figure 6:**
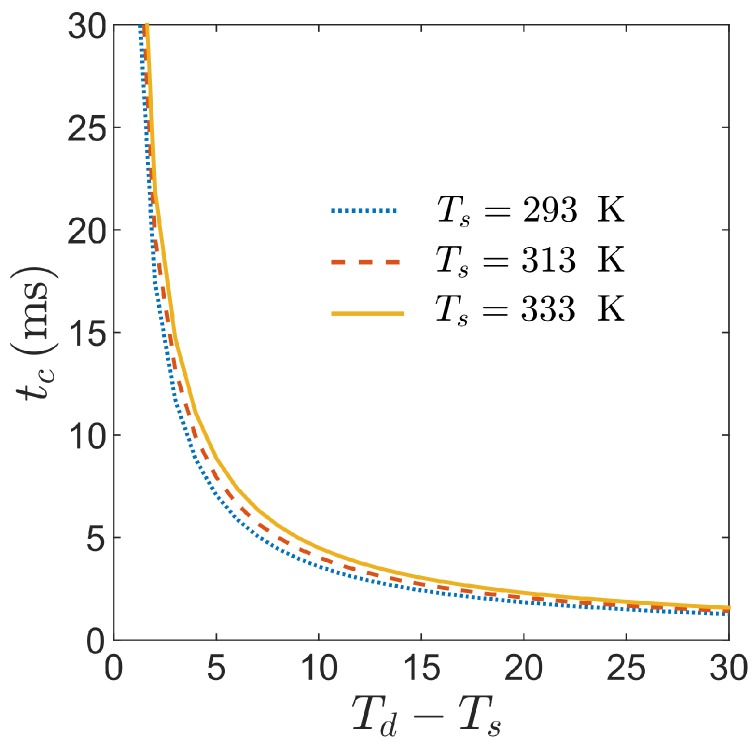
The time $t_{c}$ required for condensate to fill superhydrophobic microtexture is estimated and plotted as a function of temperature difference $T_{d}-T_{s}$ for three representative surface temperatures (curves). These values are calculated using Equation (2) assuming the properties of a water drop on a microtexture with a characteristic lengthscale of L=10μm.

Figure 6 shows plots of the condensation time, predicted from Equation (2), as a function of the temperature difference between the water drop and surface. Here, the characteristic microtexture length is L=10μm, and the different curves represent three different surface temperatures. The results show that this condensation filling timescale $t_{c}$ is significantly larger than the evaporation timescale $t_{e}$, and therefore it is reasonable to assume that the drop evaporation keeps the microtexture air fully saturated as the condensate forms. Additionally, the results illustrate the importance of the temperature difference $T_{d}-T_{s}$, rather than the temperatures themselves, in determining the condensation filling timescale $t_{c}$.

For the evaporation–condensation mechanism to trap a drop, the condensate must sufficiently fill the microtexture air before the drop bounces off of the surface. Therefore, it is natural to expect this process to depend on the relative scales of the condensation time $t_{c}$ and the drop residence time $t_{r}∼\;\sqrt{\rho_{\ell }R^{3}/\gamma }$. This ratio motivates a dimensionless grouping of parameters, which we define as β:

(3) $$\frac{t_{c}}{t_{r}}∼\beta \equiv \left(\frac{L}{R}\right)\left(\frac{\rho_{\ell }}{\rho_{v}}\right)\left(\frac{T_{sat}}{T_{sat}-T_{s}}\right){\left(\frac{\bar{R}T_{sat}\gamma }{\bar{M}H_{fg}^{2}\rho_{\ell }R}\right)}^{1/2}.$$

Here, we have dropped all dimensionless prefactors, as the focus is on the scaling relationship and the grouping of the dimensional parameters. Note that a condensation time $t_{c}$ requires $T_{sat}>T_{s}$; however, our definition for β is valid regardless of the values of $T_{s}$ and $T_{sat}$, which we have defined as $T_{sat}\equiv \left(T_{d}+T_{s}\right)/2$.

**Figure 7:**
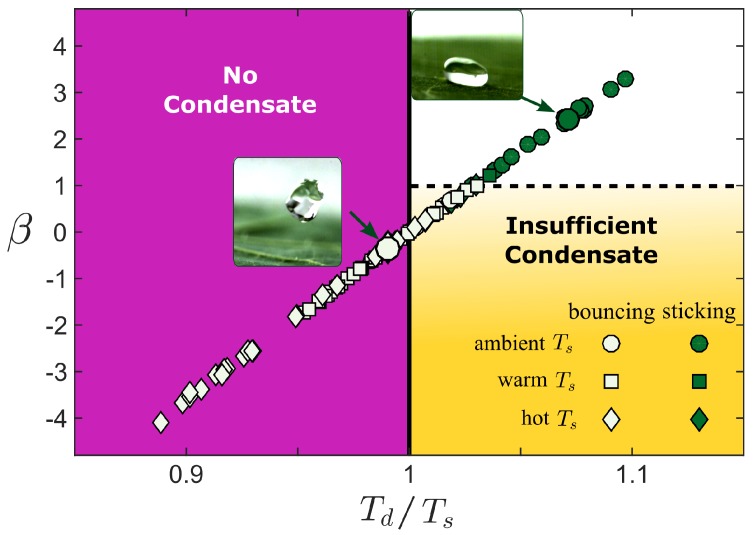
A phase plot illustrating the three condensate regimes. The experimental data from Figure 5 is replotted in terms of the temperature ratio $T_{d}/T_{s}$ and the proposed dimensionless group β (Equation (3)). Here, only data below the melting temperature $T_{m}$ is considered. When no condensate is expected to form within the microtexture (purple region), the drop is expected to bounce, whereas, when significant condensation is expected within the microtexture, it is expected to stick (white region). The finite timescale of the bounce introduces a third regime (yellow region) in which condensate develops but can be insufficient to trap the drop.

The relevance of the parameter β in the drop trapping is illustrated in Figure 7. Here, a value of β is calculated for each experimental data point in Figure 5 and plotted in terms of the ratio $T_{d}/T_{s}$ (symbols in Figure 7). Small values of β are associated with drop bouncing and large values of β are associated with drop sticking. Furthermore, the mechanics motivating the parameters suggest the data fall in one of three regimes. When $T_{d}/T_{s}<1$, the surface is hotter than the drop, and therefore even when the gap air is completely saturated, it will be below saturation directly on the texture surface so that no condensation will form, the surface will remain dry, and drops will bounce (purple region). When $T_{s}/T_{d}>1$, condensation would be expected to form and, if β is above a critical value, it would sufficiently fill the microtexture and trap the drop (white region). From the data, it appears that the critical value is around unity, although caution should be taken interpreting this value too closely, as estimates of certain parameters, such as the characteristic microtexture lengthscale of the Nasterium leaf, are less precise than others. Finally, our results indicate that if β<1, bouncing on the Nasterium leaf occur even when $T_{s}/T_{d}>1$ (yellow region). In our model, this region represents drops that bounce on superhydrophobic surfaces that are filled with insufficient condensate to transition the drop into a Wenzel state.

## 5. Conclusions

We model two potential mechanisms in which a superhydrophobic surface could trap a sufficiently hot drop within milliseconds. The first is a mechanism by which the superhydrophobic texture can be melted by the heat exchanged from the droplet during contact. The second is a mechanism by which liquid evaporates from the drop and condenses within the microtexture during contact. Because both of these mechanisms require sufficient time to develop to a scale where they adequately influence the microtexture, we highlight the importance of the residence, or contact, time of the drop to propose regimes in which these mechanisms—while present—are insufficient to prevent bouncing.

Of particular interest is the evaporation–condensation mechanism, as condensate can form under the drop whenever a drop is hotter than the superhydrophobic surface and this condensate can compromise the air pocket within the microtexture [11,12,13]. However, the experimental results in this study demonstrate that drops can bounce off of a superhydrophobic surface even when condensate is expected to form. Motivated by a balance of condensation and bouncing timescales, we propose a dimensionless group β, (Equation (3)), whose value indicates the importance of condensation as a trapping mechanism. When β is positive, condensate would be expected to nucleate; however, only for β greater than approximately 1 would trapping be expected. We anticipate that this criteria would extend to a wide range of superhydrophobic surfaces and could be relevant to a variety of applications, including the design of smart superhydrophobic surfaces that immediately trap drops that exceed a critical temperature.
